# Joint hypermobility in children with idiopathic scoliosis: SOSORT award 2011 winner

**DOI:** 10.1186/1748-7161-6-22

**Published:** 2011-10-07

**Authors:** Dariusz Czaprowski, Tomasz Kotwicki, Paulina Pawłowska, Lukasz Stoliński

**Affiliations:** 1 Faculty of Physiotherapy, Józef Rusiecki University College in Olsztyn, 10-243 Olsztyn, Bydgoska 33, Poland; 2 Department of Pediatric Orthopedics and Traumatology, University of Medical Sciences inPoznań, 61-545 Poznań, ul. 28 Czerwca nr 135, Poland; 3 Rehasport Clinic, Poznań, Poland

**Keywords:** assessment of joint hypermobility, joint laxity, idiopathic scoliosis, Beighton scale

## Abstract

**Background:**

Generalized joint hypermobility (JHM) refers to increased joint mobility with simultaneous absence of any other systemic disease. JHM involves proprioception impairment, increased frequency of pain within joints and tendency to injure soft tissues while performing physical activities. Children with idiopathic scoliosis (IS) often undergo intensive physiotherapy requiring good physical capacities. Further, some physiotherapy methods apply techniques that increase joint mobility and thus may be contraindicated.

The aim of this paper was to assess JHM prevalence in children with idiopathic scoliosis and to analyze the relationship between JHM prevalence and the clinical and radiological parameters of scoliosis. The methods of assessment of generalized joint hypermobility were also described.

**Materials and methods:**

This case-control study included 70 subjects with IS, aged 9-18 years (mean 13.2 ± 2.2), Cobb angle range 10°-53° (mean 24.3 ± 11.7), 34 presenting single curve thoracic scoliosis and 36 double curve thoracic and lumbar scoliosis. The control group included 58 children and adolescents aged 9-18 years (mean 12.6 ± 2.1) selected at random. The presence of JHM was determined using Beighton scale complemented with the questionnaire by Hakim and Grahame. The relationship between JHM and the following variables was evaluated: curve severity, axial rotation of the apical vertebra, number of curvatures (single versus double), number of vertebrae within the curvature (long versus short curves), treatment type (physiotherapy versus bracing) and age.

Statistical analysis was performed with Statistica 8.1 (StatSoft, USA). The Kolmogorov-Smirnov test, U Mann-Whitney test, Chi^2 ^test, Pearson and Spermann correlation rank were conducted. The value *p *= 0.05 was adopted as the level of significance.

**Results:**

JHM was diagnosed in more than half of the subjects with idiopathic scoliosis (51.4%), whilst in the control group it was diagnosed in only 19% of cases (*p *= 0.00015). A significantly higher JHM prevalence was observed in both girls (*p *= 0.0054) and boys (*p *= 0.017) with IS in comparison with the corresponding controls. No significant relation was found between JHM prevalence and scoliosis angular value (*p *= 0.35), apical vertebra rotation (*p *= 0.86), the number of vertebrae within curvature (*p *= 0.8), the type of applied treatment (*p *= 0.55) and the age of subjects (*p *= 0.79). JHM prevalence was found to be higher in children with single curve scoliosis than in children with double curve scoliosis (*p *= 0.03).

**Conclusions:**

JHM occurs more frequently in children with IS than in healthy sex and age matched controls. No relation of JHM with radiological parameters, treatment type and age was found. Systematically searched in IS children, JHM should be taken into account when physiotherapy is planned.

## Background

Generalized joint hypermobility (JHM) is diagnosed when the mobility of small and large joints is increased in relation to standard mobility for any given age, gender and race, and after excluding systemic diseases [1].

The data concerning prevalence of JHM among children and adolescents varies significantly, ranging from 7 to 65% [2-6]. This discrepancy seems to depend on methodological differences - the threshold values in screening tests, gender and age of subjects. The majority of authors noted the co-occurrence of JHM with the following symptoms: back pain, anterior knee pain (femoro-patellar joint pain), foot pain, flat or plano-valgus foot as well as with disturbance of posture, particularly scoliotic posture and sway back posture [2,3,7-9]. Extreme positioning of joints, very typical of JHM patients, is commonly used by children in order to enhance postural stability. Moreover, children and adolescents with JHM can suffer from shortening of breath, decreased respiratory thorax expansion and more frequent mitral valve prolapse [4,10,11]. Proprioception is also disturbed, resulting in problems with accurately determining angular joint location [12,13]. The clinical consequence of generalized joint laxity may lead to repetitive joint injuries and consequently, to joint instability, subluxation and dislocation [4].

Idiopathic scoliosis (IS) is a multiplanar spine deformation, occurring in 0.5-3.0% of adolescents [14]. In accordance with guidelines provided by SOSORT for cases of mild and moderate scoliosis, conservative treatment is recommended [15]. This consist of corrective bracing and physiotherapy. Some methods of physiotherapy used to treat scoliotic children, include exercises that aim at increasing the range of spinal mobility to achieve curve correction [16-19]. It may be supposed that these exercises result in increased spinal mobility. Moreover, some therapeutic systems rely on proprioception to increase patient's ability to a sense of joint position [16,20,21]. There is a dearth of in-depth reports on JHM prevalence in children with idiopathic scoliosis.

The aim of this study was to assess joint hypermobility occurrence in children with idiopathic scoliosis and to analyze the relationship between the JHM and the clinical and radiological parameters of scoliosis.

## Materials and methods

### Subjects

The case-control study included 70 subjects aged 9-18 years (mean 13.2 ± 2.2) with idiopathic scoliosis. The criteria for inclusion to the study group were the following: diagnosis of idiopathic scoliosis on antero-posterior radiogram in accordance with SRS criteria (Cobb angle > 10° with rotation), age range 9-18 years, absence of systemic diseases related to JHM (Ehlers-Danlos, Down, Marfan, Larsen) and participation consent. The control group consisted of 58 subjects (mean age 12.6 ± 2.1 years) randomly chosen according to the inclusion criteria: age range 9-18 years, less than 5° of angle of trunk rotation as measured with Bunnell scoliometer, absence of systemic diseases and participation consent. Approval of the local ethical committee was obtained. The basic data for both groups is presented in Table 1.

**Table 1:** Parameters of the study and the control group

	Study group n = 70 average (SD)	Control group n = 58 average (SD)	*p*
Age (years)	13.2 (2.2)	12.6 (2.1)	0.08
Height (m)	1.59 (0.1)	1.56 (0.1)	0.2
Weight (kg)	49.5 (13.0)	47.4 (14.8)	0.7
BMI (kgm^-2^)	19.2 (3.4)	18.8 (3.5)	0.99
Cobb (°)	24.3 (11.7)	-	-

Additionally, the comparability between the study group and the control group was checked separately for the girls (n = 92) and boys (n = 36). The girls from the study group were significantly taller than the girls from the control group (*p *= 0.02), however, the groups were comparable in respect to age (*p *= 0.06), weight (*p *= 0.2) and BMI (*p *= 0.4). Boys from the study group were comparable to boys from the control group in respect to age (*p *= 0.1), height (*p *= 0.1), weight (*p *= 0.3) and BMI (*p *= 0.8).

Within the study group, the Cobb angle range was 10° to 53°. In 34 cases, a single-curve thoracic scoliosis was present, while in the remaining 36 cases - a double-curve thoracic and lumbar scoliosis. The average number of vertebrae forming single-curve scoliosis was 6.5 vertebrae (range 4-10). The value of apical vertebra rotation (AVR) was determined using the Cobb method [22]. 47 subjects were treated exclusively with physiotherapy, while the remaining 23 subjects received both physiotherapy and treatment with Cheneau brace.

### Instrumentation

**Figure 1:**
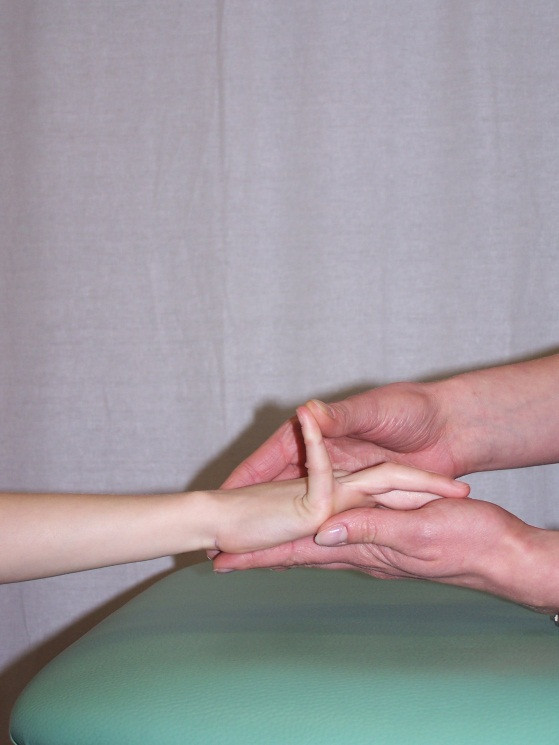
**Extension of the MCP joint of the fifth finger**.

**Figure 2:**
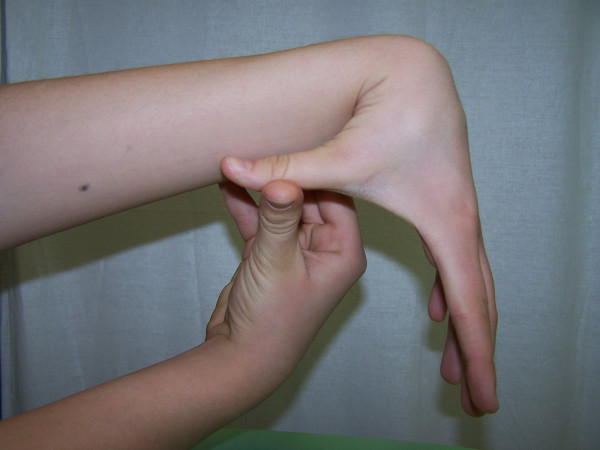
**Abduction of the thumb to the forearm**.

**Figure 3:**
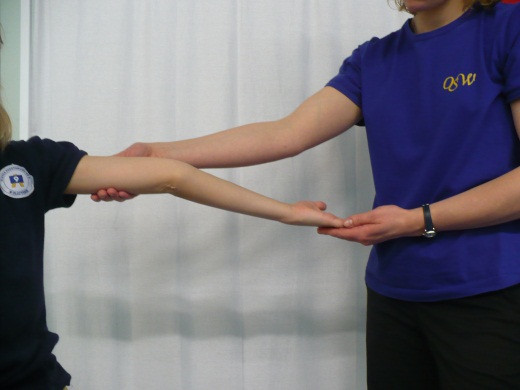
**Elbow hyperextension**.

**Figure 4:**
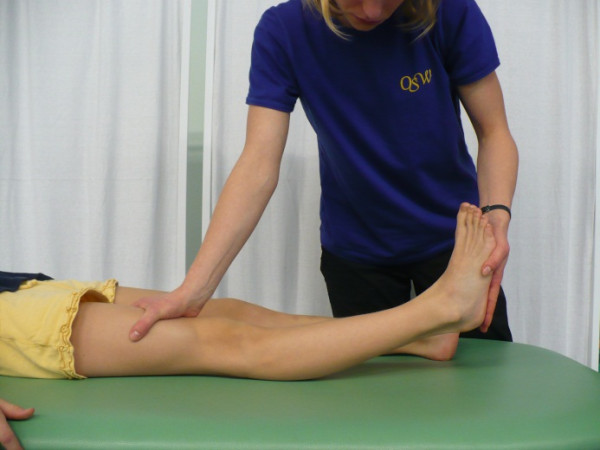
**Knee hyperextension**.

**Figure 5:**
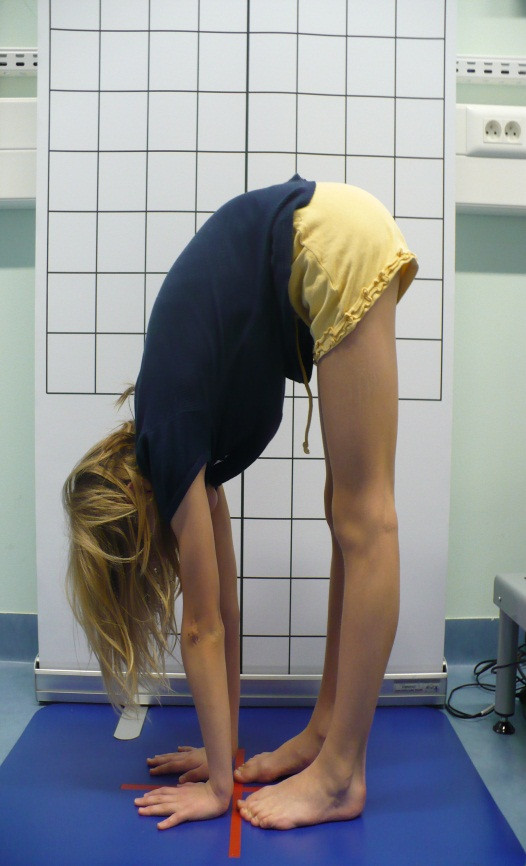
**Touching the floor with the palms of the hands**.

A nine-degree Beighton scale was used to determine the occurrence of generalized joint hypermobility [23] (Figure 1, 2, 3, 4, 5). In order to assess the range of joint mobility, a set of goniometers was used (Baseline, USA).

Additionally, a five-part questionnaire by Hakim and Graham was used [24] (Table 2). Obtaining 4 or more points on Beighton scale and simultaneously at least 2 points in the ques-tionnaire by Hakim and Graham was adopted as a criterion for JHM diagnosis.

**Table 2:** A five-part questionnaire for identifying joint hypermobility [24]

1. Can you now (or could you ever) place your hands flat on the floor without bending your knees?

2. Can you now (or could you ever) bend your thumb to touch your forearm?

3. As a child did you amuse your friends by contorting your body into strange shapes or could you do the splits?

4. As a child or teenager did your shoulder or kneecap dislocate on more than one occasion?

5. Do you consider yourself double-jointed?

The frequency of occurrence of the JHM was compared between the study and control groups. The same analysis was also performed separately for girls and boys. JHM occurrence was compared between mild (Cobb 10°-24°), moderate or severe (Cobb ≥25°) idiopathic scoliosis. Correlation between the Cobb angle and the number of points on the Beighton scale was checked. The relation of JHM occurrence to the following parameters was tested: (1) the apical vertebra rotation quantified according to Cobb as + versus ++ versus +++, (2) number of curvatures - single versus double, (3) number of vertebrae within the single scoliosis curvature: above versus below the mean of 6.5 vertebrae, (4) type of management: physiotherapy alone versus physiotherapy and corrective bracing, (5) the age of patient: above or below the mean of 13.2 years. Correlation between the number of points in Beighton scale and age as well as between Beighton scale and the number of vertebrae included in the primary curvature was calculated.

Statistical analysis was performed with Statistica 8.1 (StatSoft, USA). The Kolmogorov-Smirnov test was initially applied to check normal distribution. The U Mann-Whitney test, Chi^2 ^test, Pearson and Spermann correlation rank were also conducted to. The value *p *= 0.05 was adopted as the level of significance.

## Results

JHM was diagnosed in more than half the children with idiopathic scoliosis (51.4%), whilst in the control group, it was diagnosed only in 19.0% of cases (*p *= 0.00015) (Table 3).

**Table 3:** Prevalence of joint hypermobility (JHM) in the study and the control group

	Study group n = 70 (100.0%)	Control group n = 58 (100.0%)
JHM present - n (%)	36 (51.4)	11 (19.0)
JHM absent - n (%)	34 (48.6)	47 (81.0)

*p *value	**0.00015**	

Both in the group of girls (Table 4) and boys (Table 5) with IS, significantly higher frequency of JHM was observed in comparison with the corresponding control groups.

**Table 4:** Difference in prevalence of joint hypermobility (JHM) between girls from the study and from the control group

	Study group - girls n = 59 (100.0%)	Control group - girls n = 33 (100.0%)
JHM present - n (%)	30 (50.8)	7 (21.0)
JHM absent - n (%)	29 (49.2)	26 (79.0)

*p *value	**0.0054**	

**Table 5:** Difference in prevalence of joint hypermobility (JHM) between boys from the study and from the control group

	Study group - boys n = 11 (100.0%)	Control group - boys n = 25 (100.0%)
JHM present - n (%)	6 (54.5)	4 (16.0)
JHM absent - n (%)	5 (45.5)	21 (84.0)

*p *value	**0.017**	

JHM occurred in 56.1% children with mild scoliosis (10-24° Cobb). In children with scoliosis of 25° and above, the percentage of JHM was 44.8%, difference not significant, *p *= 0.35 (Table 6). The calculation of correlation between the angular value of curvature (Cobb degrees) and the number of points in Beighton scale failed to show any significant correlation (*R *= -0.099; *p *= 0.4).

**Table 6:** Prevalence of joint hypermobility (JHM) in relation to curve angle

	Cobb 10°-24° n = 41 (100.0%)	Cobb ≥25° n = 29 (100.0%)
JHM present - n (%)	23 (56.1)	13 (44.8)
JHM absent - n (%)	18 (43.9)	16 (55.2)

*p *value	0.35	

No relation between the apical vertebra axial rotation and JHM frequency was found (*p *= 0.86) (Table 7).

**Table 7:** Prevalence of joint hypermobility (JHM) in subjects with apical vertebra axial rotation (AVR) quantified according to Cobb method as: AVR +, AVR ++ or AVR +++

	AVR + n = 30 (100.0%)	AVR ++ n = 22 (100.0%)	AVR +++ n = 18 (100.0%)
JHM present - n (%)	14 (46.7)	12 (54.5)	8 (44.4)
JHM absent - n (%)	16 (53.3)	10 (45.5)	10 (55.6)

*p *value	0.86		

Joint hypermobility occurred in 64.7% children with single-curve scoliosis, whilst in children with double-curve scoliosis, the percentage was below 39% which was significantly different (***p *= 0.03**) (Table 8).

**Table 8:** Determining the level of diversity in joint hypermobility (JHM) prevalence in subjects with single-curve versus double-curve scoliosis

	Single-curve n = 34 (100.0%)	Double-curve n = 36 (100.0%)
JHM present - n (%)	22 (64.7)	14 (38.9)
JHM absent - n (%)	12 (35.3)	22 (61.1)

*p *value	**0.03**	

No statistical relationship was observed between JHM prevalence and the length of single-curve scoliosis. Comparison of JHM percentage in children with the length of scoliosis below and above the accepted average proved to be statistically insignificant (*p *= 0.8) (Table 9), as did the correlation between the number of vertebra included in scoliotic deformation and the number of points obtained on Beighton scale (*R *= -0.043, *p *= 0.8).

**Table 9:** Determining the level of differences in JHM prevalence in subjects with scoliosis length below (< 6

	Group < 6.5 n = 18 (100.0%)	Group > 6.5 n = 16 (100.0%)
JHM present n (%)	12 (66.7)	10 (62.5)

JHM absent (%)	6 (33.3)	6 (37.5)

*p *value	0.8	

Joint hypermobility was observed in 56.5% of children treated by both physiotherapy and Cheneau brace in comparison to 48.9% of children treated only by physiotherapy, difference not significant (*p *= 0.55) (Table 10).

**Table 10:** Assessment of joint hypermobility (JHM) prevalence in the group of subjects treated only with physiotherapy versus treated with physiotherapy and Cheneau brace

	Physiotherapy n = 47 (100.0%)	Physiotherapy and bracing n = 23 (100.0%)
JHM present - n (%)	23 (48.9)	13 (56.5)
JHM absent - n (%)	24 (51.1)	10 (43.5)

*p *value	0.55	

Although the literature data shows decreasing JHM prevalence with age [21], this study did not show significant differences in the frequency of JHM occurrence in children with scoliosis aged 13.2 years or younger versus children aged over 13.2 (*p *= 0.79) (Table 11). However, the number of points on Beighton scale tended to decrease with age (*R *= -0.268; ***p *= 0.02**).

**Table 11:** Comparison of joint hypermobility (JHM) prevalence in group of subjects with scoliosis aged below versus above the average of 13

	< 13.2 n = 32 (100.0%)	> 13.2 n = 38 (100.0%)
JHM present - n (%)	17 (53.1)	19 (50.0)
JHM absent - n (%)	15 (46.9)	19 (50.0)

*p *value	0.79	

## Discussion

JHM is not considered to be a specific disease, but rather a phenomenon concerning the musculo-skeletal system of an individual subject [4]. The borderline between constitutional generalized joint hypermobility versus pathological skin and joint laxity (connective tissue disorders) is not always easily defined. In the classification of children's diseases adopted by pediatricians, patients with generalized joint hypermobility are reported to have a Benign Joint Hypermobility Syndrome (BJHS) [1,25]. The term was introduced to emphasize the differences between BJHS and HDCT - Hereditary Disorders of Connective Tissue [25]. The latter, which pertains to innate disorders in the connective tissue, is exemplified by Marfan syndrome, Larsen syndrome, Ehlers-Danlos syndrome or osteogenesis imperfecta [1,26,27]. These diseases represent well described clinical entities, however, differential diagnosis of some border conditions, for example of benign forms of Ehlers-Danlos syndrome, would require genetic examination. Nevertheless, in screening and in everyday practice, the clinical examination remains the means to assess both benign constitutional joint hypermobility and pathological soft tissue laxity. It is important to remember that the latter is expressed within the skin; the assessment of the skin fold and skin laxity being an important part of the examination.

This study showed that JHM occurs more frequently in patients with IS, in comparison with healthy controls. This regularity applies to both girls and boys. No relation between the frequency of JHM occurrence and the angular value of scoliotic curvature, apical vertebra rotation, length of scoliosis or applied conservative treatment was observed. No correlation between the number of Beighton scale points and the angular value of curvature was found. This study suggests that even if children with IS are more prone to present joint hypermobility, this is not associated with radiological parameters of scoliosis. JHM rate was significantly higher in the group of children with single curve scoliosis in comparison to children with double curve scoliosis (*p *= 0.03). This finding requires further studies, to exclude whether it represented just a statistical phenomenon; it seems unjustified to speculate on cause-result relation at this point.

Joint mobility range decreases with age. It is at its highest point just after birth, after which it gradually decreases, most rapidly in the childhood period [4,5,7,23,25]. This natural tendency for JHM frequency to diminish with age was not confirmed by our study, as JHM did not differ significantly in children aged below versus those aged above the average of 13.2 years (*p *= 0.79). However, in children with IS, the number of Beighton scale points tended to decrease with age (*p *= 0.02). The results indicate that while JHM frequency does not decrease with age of children with IS, the intensity of clinical signs seems to diminish.

### Methods of assessment of generalized joint hypermobility

Different authors apply various names to define increased joint mobility, despite using similar diagnostic scales. The following exemplifies this variety: Joint hypermobility (JHM), Joint hypermobility syndrome - JHS, Benign hypermobility syndrome (BHS), Benign joint hypermobility syndrome (BJHS) or Joint laxity [2-8,12,25-31]. This lack of consistency is an obstacle to direct comparison of results [4]. Gedalia et al., with the use of Beighton scale, reported that JHM occurs in 12% of American pupils aged 5-17, where in 18% of girls and in 7% of boys [2]. The study by Vougiouka et al. assessing right limbs and lumbar spine flexion proved that Benign Hypermobility Syndrome occurs in 8.78% of children aged 5-14 [3]. The authors emphasize that BHS occurs in a significantly smaller percentage of boys than girls (7.1% versus 10.7%). Hakim and Grahame analyzed the results of 7 examinations conducted on children aged 11-17 [4]. Their analysis shows that JHM occurs in approximately 10-15% of boys and 20-40% of girls. Observations made with the use of Beighton scale in Brazilian children aged 4-7 indicated that as many as 64.6% of children in that group presented JHM [5]. Similar figures were obtained by de Inocencio Arocena et al. (Beighton scale), who confirmed JHM occurrence in 55% of children aged 4-14 [6]. It seems that important differences between publications result basically from the diverse methodologies used.

### Beighton scale

The most frequent method used in clinical screening is Beighton 9-point scale [4,23,32,33]. It consists of exclusively assessing joint mobility: extension of the fifth MPC joint to 90°, thumb abduction to front forearm, hyperextension of elbow joint above 10°, hyperextension of knee joint above 10° as well as capability to stand bend and place one's palms flat on the ground. Each hypermobile joint gets one point. To diagnose JHM, at least 4 points have to be obtained [4,23,34].

### Carter and Wilkinson method

**Figure 6:**
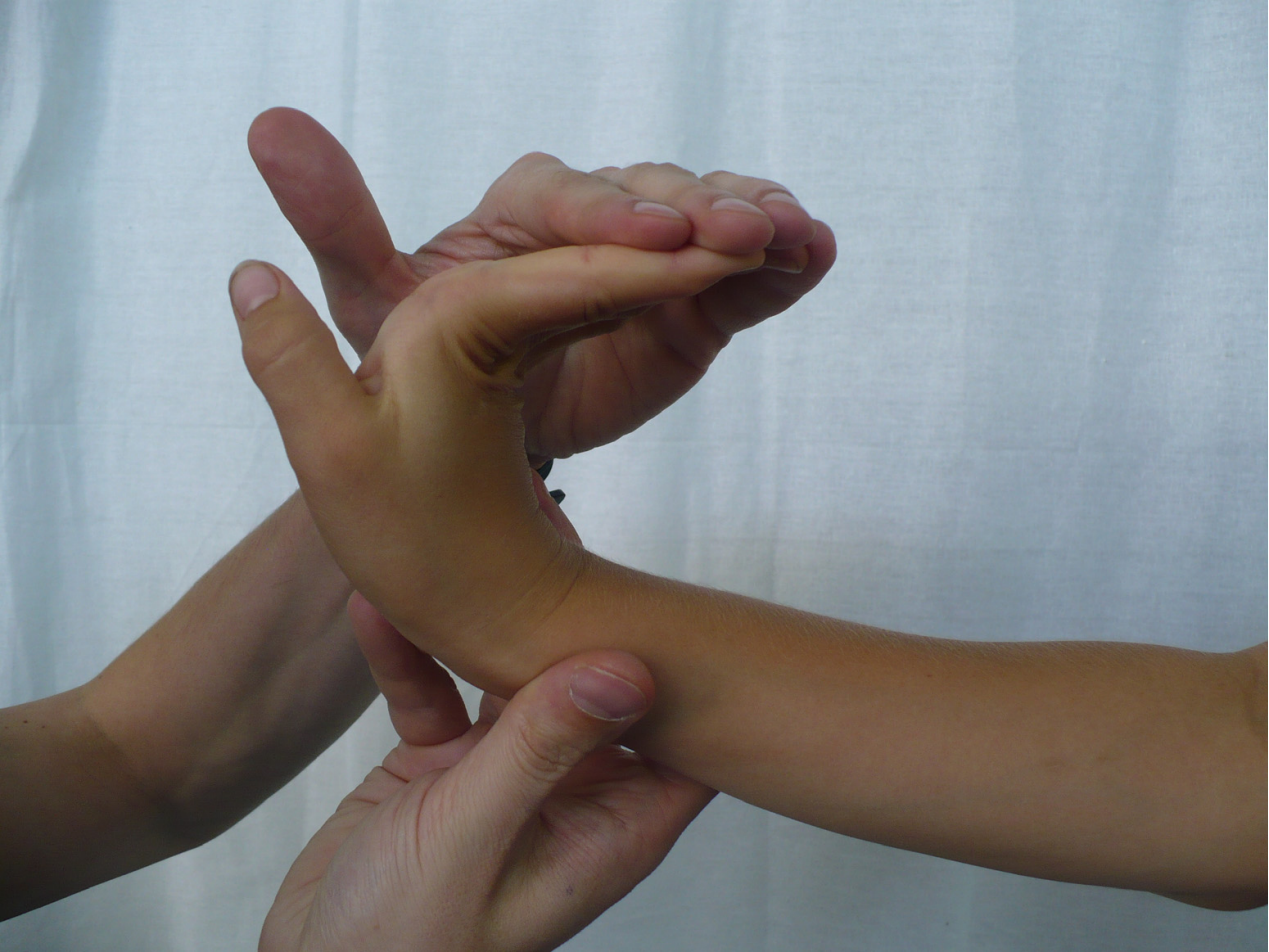
**Passive hyperextension of all MCPs of the II-V fingers to a position parallel to the extensor aspect of the forearm**.

**Figure 7:**
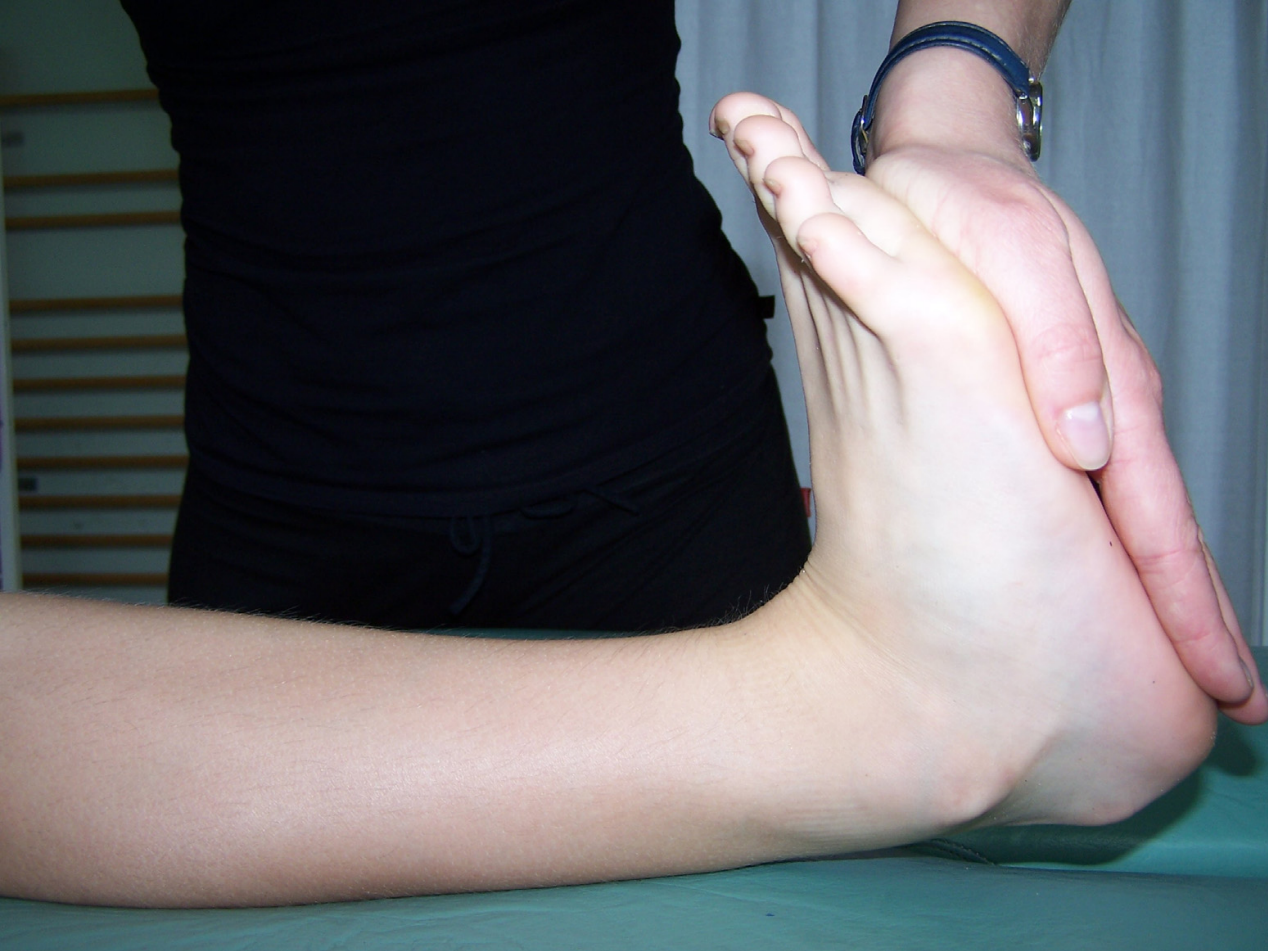
**Ankle dorsiflexion**.

Carter and Wilkinson published a method of assessment of generalized joint hypermobility about a decade before Beighton score was introduced [29-31]. Their scale takes into account similar articulations: the thumb, elbow and knee joints. The first difference from Beighton scale concerns the assessment of passive hyperextension of all four II-V fingers to a position parallel to the forearm extensor aspect (Figure 6), instead of the assessment of the fifth finger only. The second difference involves assessing the range of ankle dorsiflexion beyond 45° in the Carter and Wilkinson's method (Figure 7), instead of assessing the ability to touch the ground with one's palms as adopted by Beighton. Both scales are considered to be reliable in the assessment of joint hypermobility [30]. It is also important to notice that ankle dorsiflexion beyond 45° may be limited even in hypermobile subjects by relative shortening of the triceps muscle which is quite a common condition in a growing population. On the other hand, touching the floor with one's hands, as proposed by Beighton, can be executed by subjects who are not hypermobile but who have experienced intensive stretching of the hamstring muscles.

### Marshall test

**Figure 8:**
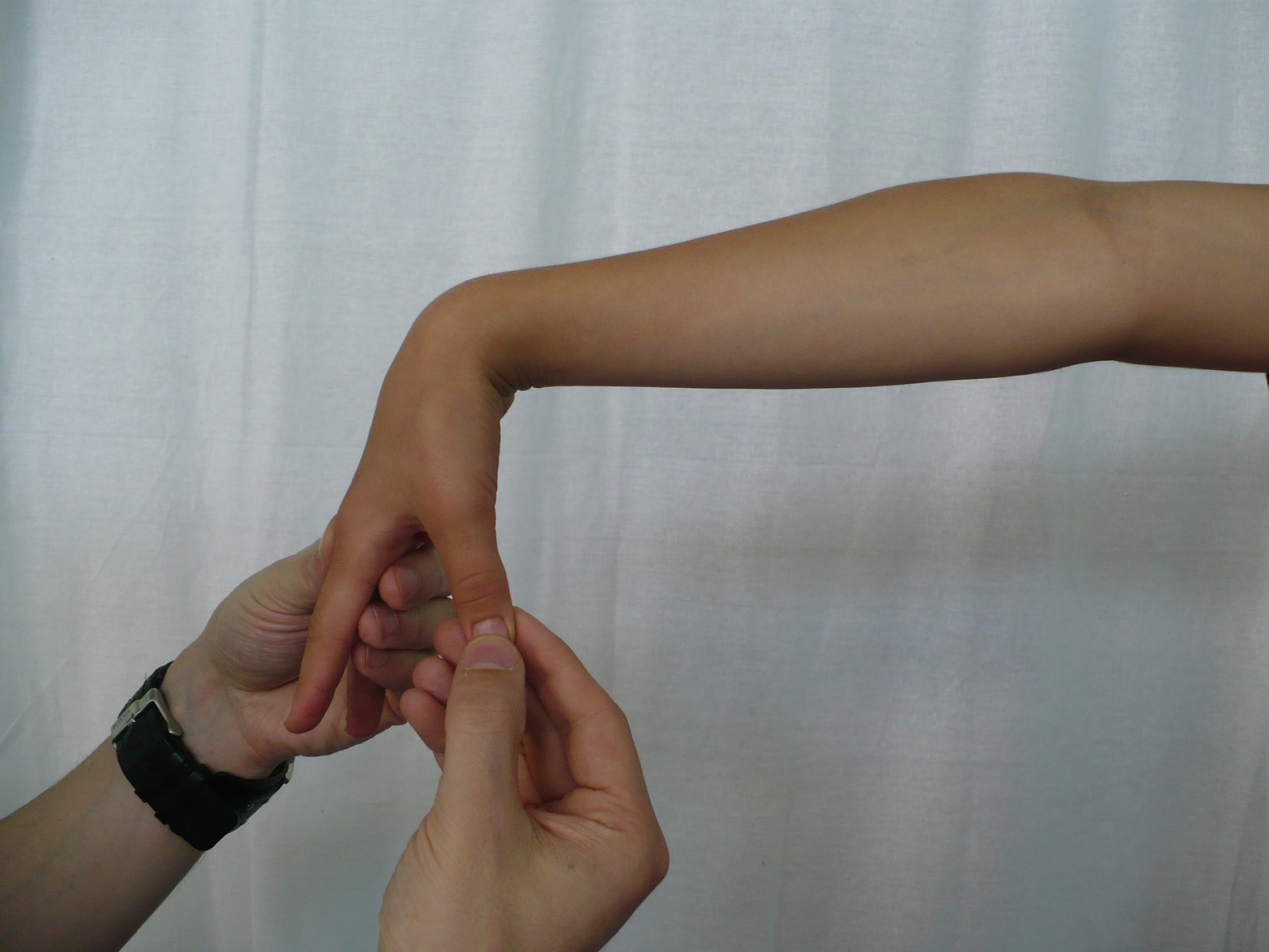
**I - < 45° of passive thumb abduction**.

**Figure 9:**
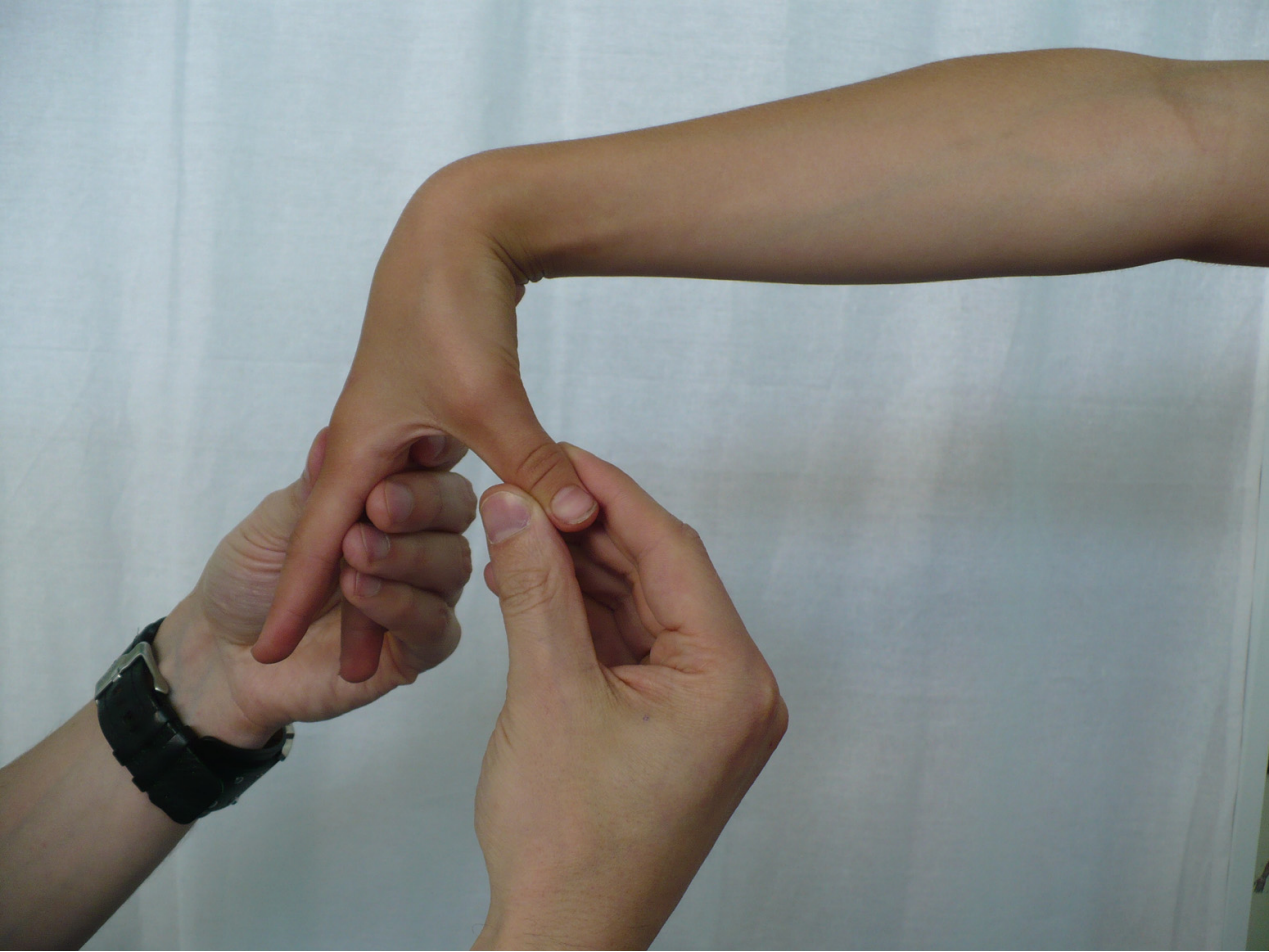
**II - 45° of thumb abduction**.

**Figure 10:**
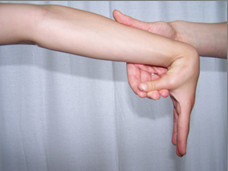
**III - 90° of thumb abduction**.

**Figure 11:**
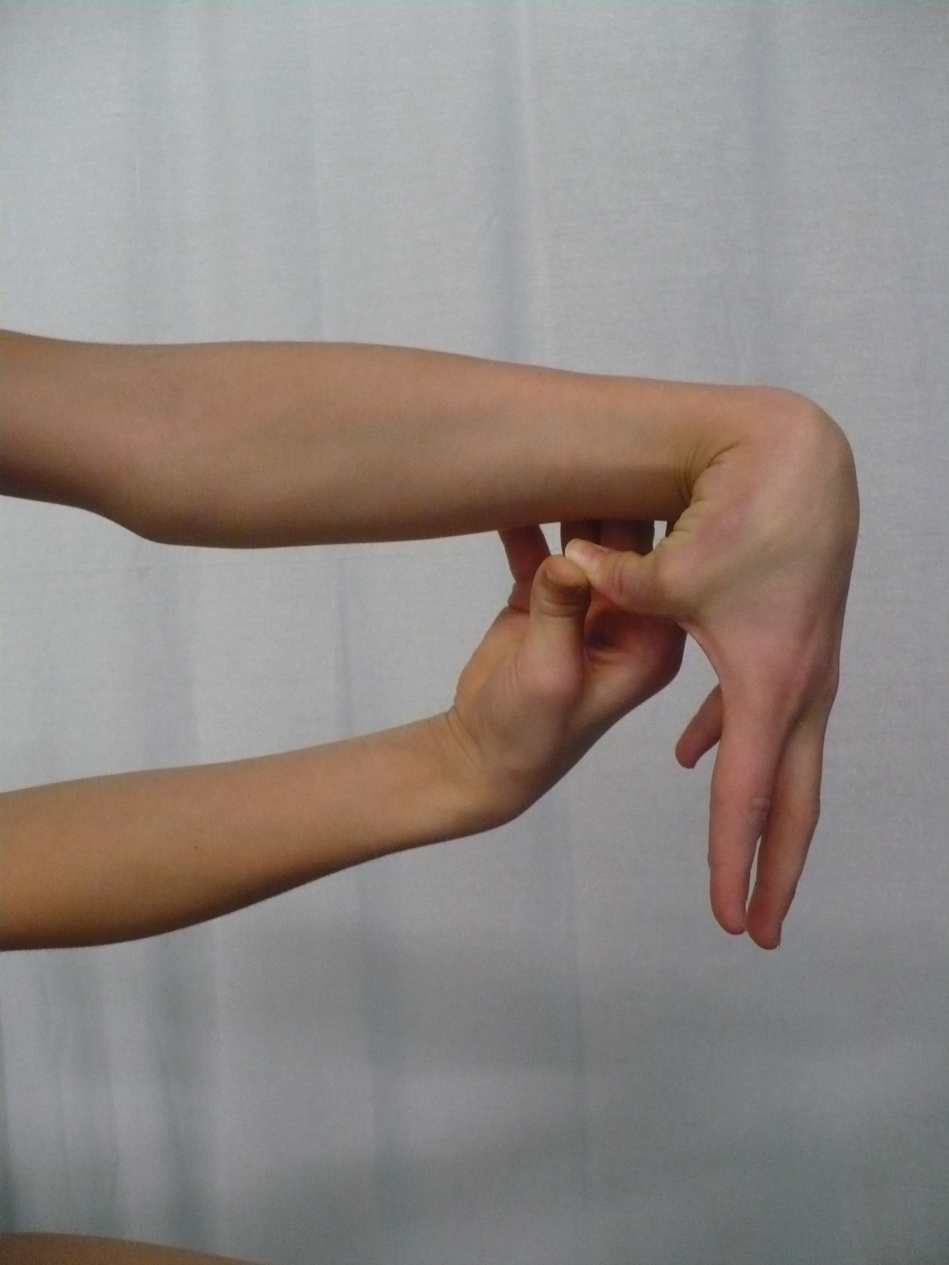
**IV - 135° of thumb abduction**.

**Figure 12:**
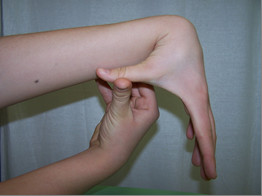
**V - Thumb opposed to the forearm (as in the Beighton scale)**.

Marshall test is another method of assessing joint hypermobility based on the thumb motion range measured in the forearm direction [35]. The following figures show scores obtained during this test: I - < 45° of passive thumb abduction (Figure 8); II - 45° of abduction (Figure 9); III - 90° of abduction (Figure 10); IV - 135° of abduction (Figure 11), and V - thumb can be opposed to the forearm (Figure 12). Marshall test is widely used for its simplicity. The disadvantage of this test is the fact that it focuses only on one joint.

### Bulbena scale

Bulbena scale is a 10-point scale used to assess generalized joint hypermobility [36]. It takes into consideration the signs in upper extremities, lower extremities and skin. The above described methods are summarized in Table 12.

**Table 12:** Comparison of Beighton, Carter and Wilkinson, Bulbena and Marshall scales for diagnosis of joint hypermobility

Criterion	Beighton [4,23,34]	Carter and Wilkinson [29]	Bulbena [36]	**Marshall **[35]
Thumb abduction	1^a^	1	1	Grade I through V
Elbow hyperextension > 10°	1^a^	1	1	
Fifth finger extension > 90° in MCP joint	1^a^	1 passive hyperextension of II-V fingers parallel to the forearm	1	
Knee hyperextension > 10°	1^a^	1		
Foot dorsiflexion		1 (> 45°)	1 (> 20°)	
Palms flat on floor	1			
Passive shoulder external rotation > 85°			1	
Knee flexion allows the heel to make contact with the buttocks			1	
Passive shift of the patella to the lateral side of the tibia				
Passive hip abduction > 85°			1	
Hyperextension of MTP joints of the first finger > 90°			1	
Appearance of ecchymoses after hardly noticed minimal trauma			1	

Total score	9	5	10	I-V

Number of point for diagnosis	4 [4,6,33,34] 7 [32]	3 [29]	female ≥5, male ≥4 [36]	

^a ^1 point per each side

### Threshold to diagnose JHM

Apart from various methods used to assess generalized joint hypermobility, within a given method, different cut-off values have been proposed. For the purposes of diagnosing JHM in children, Lamari et al. adopted 3 of the 9 points values from Beighton scale [5]. A similar threshold was adopted by Gedalia et al. [2]. According to Grahame and Bird 87% of British rheumatologists consider that at least 3 hypermobile joint suffice to diagnose JHM [37]. However, Smits-Engelsman et al. suggests that the minimal threshold value should be at least 7 points in the 9-point scale [32]. In 1998, criteria from Brighton were introduced to diagnose BJHS, comprising the so-called major and minor criteria. However, the Brighton scale requires specific examinations which are difficult in screening conditions, for example rectal prolapse or joint inflammation [4,28]. Hakim and Grahame as well as Hakim and Sahota suggest that the diagnosis of JHM should be supplemented with a 5-point questionnaire [24,34]. It would allow a fast clinical overview as its questions refer to symptoms observed both at present and in the past, thus accounting for mobility changes that occur with age. A positive answer to two or more questions would indicate hypermobility with sensitivity of 80-85% and specificity of 80-90% [4,24,34].

The authors of this study used a 9-point Beighton scale to assess JHM prevalence with the threshold level of minimum 4 points, supplemented with a 5-part questionnaire to avoid false positive results, as suggested by Hakim and Grahame [23,24].

The studies determining the prevalence of JHM usually refer to healthy population of children and adolescents [2,3,5,6,38,39]. The second group of papers compares occurrence of accompanying symptoms and signs in children with JHM versus their prevalence in the control group [4,7-9,40,41]. The authors, however, are not aware of any reports on JHM prevalence in children suffering from idiopathic scoliosis. Binns found de-creased thumb-to-forearm distance measured during passive apposition in Chinese patients with adolescent idi-opathic scoliosis [42]. This suggests that they are relatively hyperlax; however, the study was based solely on thumb mobility.

The issue of JHM in idiopathic scoliosis seems clinically important as these children may be subjected to intensive physiotherapy according to various approaches ("schools"). These therapeutic activities include, for example, flexibility exercises, passive stretching exercises, as well as proprioception exercises [16-21,43]. Thus, it seems crucial to assess JHM prevalence in these patients. According to Keer and Grahame, physiotherapy is a mainstay of musculoskeletal aspects of JHS treatment [44]. However, those authors emphasize the fact that patients often complain about physiotherapy, claiming that their condition seems to have deteriorated, especially when their hypermobile joints are not handled with due care while treated manually. Moreover, physiotherapists often admit that their knowledge about JHM patients is not sufficient [44]. Such knowledge has great significance since it is typical of JHM patients to be particularly vulnerable to soft tissue injuries which occur during physical exercises. Additionally the increased sensitivity of pain receptors as well as recurrent micro injuries can increase the risk of joint injuries [4,41].

The results indicate significantly higher JHM prevalence in children with idiopathic scoliosis, supported by the commonly recognized negative impact of this impairment on the locomotor system of children treated with physiotherapy. Assessing JHM needs to be taken into consideration when planning physiotherapy for IS children. This is even more true given the fact that JHM diagnosis is not time consuming and is based on screening tests that are easy to perform.

## Conclusions

1. Joint hypermobility appears more often in children with idiopathic scoliosis than in healthy age and sex matched controls.

2. There was no relation of joint hypermobility prevalence with the Cobb angle value, Cobb apical vertebra axial rotation, length of scoliosis or treatment type.

3. Joint hypermobility should be systematically assessed in IS children and when found, taken into account when physiotherapy is planned.

## List of abbreviations

JHM: Joint hypermobility; JHS: Joint hypermobility syndrome; BHS: Benign hypermobility syndrome; BJHS: Benign joint hypermobility syndrome; IS: Idiopathic scoliosis; AVR: Apical vertebral rotation; MCP: Metacarpophalangeal joint; MTP: Metatarsophalangeal joint

## Competing interests

The authors declare that they have no competing interests.

## Authors' contributions

DC conceived the study, collected and interpreted the data and drafted the manuscript. TK participated in the design of the study, interpreted the data and drafted the manuscript. PP collected the data and helped to draft the manuscript. LS helped to the draft the manuscript. All the authors read and approved the final manuscript.
